# Posterior Tibial Nerve Stimulation for Overactive Bladder: Mechanism, Classification, and Management Outlines

**DOI:** 10.1155/2022/2700227

**Published:** 2022-03-16

**Authors:** Abdullah Al-Danakh, Mohammed Safi, Mohammed Alradhi, Marwan Almoiliqy, Qiwei Chen, Murad Al-Nusaif, Xuehan Yang, Aisha Al-Dherasi, Xinqing Zhu, Deyong Yang

**Affiliations:** ^1^Department of Urology, First Affiliated Hospital of Dalian Medical University, Dalian 116021, China; ^2^Department of Oncology, First Affiliated Hospital of Dalian Medical University, Dalian 116021, China; ^3^Department of Urology, Second Affiliated Hospital of Dalian Medical University, Dalian 116021, China; ^4^Department of Pharmacology, Pharmaceutical College, Dalian Medical University, Dalian 116044, China; ^5^Department of Neurology, First Affiliated Hospital, Dalian Medical University, Dalian 116021, China; ^6^Department of Biochemistry, Faculty of Science, Ibb University, Ibb, Yemen; ^7^Healinghands (Dalian) Clinic, Dalian, Liaoning, China

## Abstract

*Purpose of the Review*. Posterior tibial nerve stimulation (PTNS) techniques have dramatically grown after approval to manage overactive bladder (OAB). The present review will focus on the most current data on PTNS types (percutaneous, transcutaneous, and implant) and their mechanism of action, safety, efficacy, advantages, drawbacks, limitation, and clinical applications. *Recent Findings*. The present review described the recent studies that addressed the tibial nerve stimulation role in OAB management. BlueWind RENOVA system, Bioness StimRouter, and eCoin are examples of emerging technologies that have evolved from interval sessions (percutaneous PTNS and transcutaneous PTNS) to continuous stimulation (implants). These can be efficiently managed at home by patients with minimum burden on the health system and fewer visits, especially in the COVID-19 pandemic. *Summary*. Our review shows that the tibial nerve stimulation advancements in OAB treatment have been rapidly increasing over the recent years. It is minimally invasive and effective, similar to sacral nerve stimulation (SNM), but less aggressive. Implantable PTNS has been promised in terms of efficacy, safety, and high acceptance rate. However, evidence is still limited to short-term trials, and tolerability, method, and drawbacks remain challenges.

## 1. Introduction

The International Continence Society (ICS) defined an overactive bladder as a syndrome characterized by “urinary urgency, with or without frequency and nocturia, with or without Urgency Urinary Incontinence (UUI), in the absence of urinary tract infection or other obvious pathology” [1, 2].

It is a prevalent problem affecting one in every five adults aged over 40 years, with women being impacted more than men [3–5]. Patients with OAB cost the US healthcare system two and half times as much as equivalent patients without OAB [6,7]. Despite the detrimental effect on work, daily living, sexual function, and negative impact on the quality of life (QoL), OAB patients seeking treatment for their symptoms have been low in recent years because of its invasiveness such as surgery [8–10].

Pharmacotherapy plays an essential role if OAB patients do not respond to behavioral and pelvic floor therapies. However, low efficacy, adverse events, and costs limit its use in large proportions of OAB patients [11–13]. Neuromodulation techniques such as sacral nerve stimulation (SNM) and PTNS effectively treat OAB. In the past, unresponsive OAB cases were treated with invasive surgical procedures such as bladder augmentation, detrusor myomectomy, and bladder denervation, associated with extended recovery times and long-term morbidity [14].

Neuromodulation, in theory, should be minimally invasive, simple to use, not cause embarrassment to the patient's private area, long-lasting, and cost-effective. [15] However, SNM was regarded as the gold standard of third-line treatment for OAB patients, but it has achieved minor advances since decades ago [16]. SNM device size, cost, radiation exposure to implant its lead near a sacral nerve, and two surgeries are the main shortcomings [17]. Furthermore, short-term and long-term complications were reported about 30-40% that require removal and replacement [18]. PTNS approved for OAB patients' treatment shows high technology progress, cost-effectiveness, and sustainability with fewer adverse events than SNM [17].

This narrative review will summarize the history, classifications, mechanism of action, and clinical application of PTNS in the treatment of OAB and the most recent advances in PTNS.

## 2. History of PTNS

By the 18th century, several electrostatic exposure devices were invented, and the unique machine, namely “electric heating developed in the 19th century,” was used to treat patients with electrical diseases [19]. In the 18th century, electrostatic generators helped popularize electrotherapy, but it declined by the late 19th century due to the increasing use of pharmacological treatment [20]. In 1954, Mr. Brown advocated the routine use of nerve stimulators to control the involuntary application of curarization in surgical patients [21]. In the 1970s, the National Institutes of Health (NIH) attempted to obtain coordinated urination through electrical stimulation but did not achieve it, but intermittent urination was achieved. Schimidt et al., in 1989, performed an SNM animal experiment to control urination in neurogenic void dysfunction, and it was the first successful SNM experience [22]. SNM was approved as an OAB therapy by the United States Food and Drug Administration (FDA) in 1997. PTNS was introduced in 1999 and approved as a treatment method for this chronic issue by the FDA in 2000 and the National Institute for Health and Clinical Excellence (NICE) in 2009 [23]. Like traditional Chinese acupuncture used to manage many diseases in Chinese medicine, McGuire was the first to show electrical stimulation of the tibial nerve, resulting in decreased detrusor overactivity [24]. Stoller defined PTNS as a lower urinary tract neuromodulation technique that used electrical stimulation of the posterior tibial nerve to treat OAB syndrome in the late 1990s [25].

## 3. Micturition Reflex and Neuromodulation Mechanism

**Figure 1:**
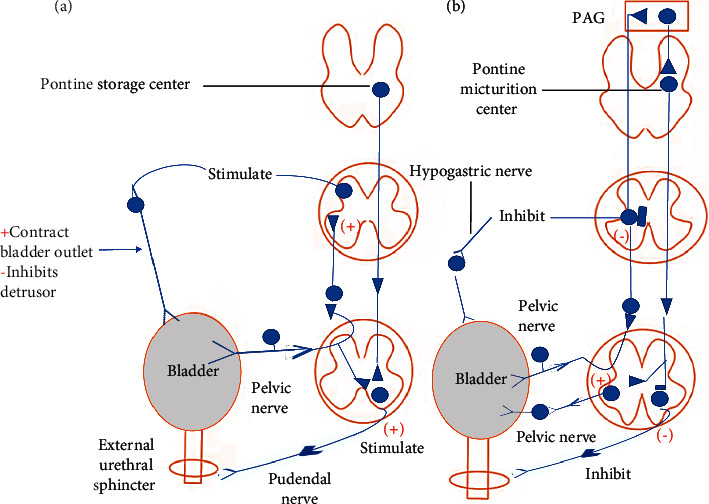
The brain networks that regulate continence and micturition are depicted. Urine storage reflexes. (a). Urine storage reflexes. (b). Urination (or voiding) reflexes the pontine micturition center (PAG).

Micturition is regulated by micturition centers in the cortical, pontine, and spinal cord. When we urinate in small amounts, the cortical micturition center makes the decision. As a result, it has the potential to either facilitate or obstruct [26]. Anxiety can increase central facilitation, which can lead to frequency, and in extreme anxiety situations, frequency and even UUI are common but occur only during the daytime, unlike the frequency caused by urothelium irritation, which occurs day and night [27]. Along with being unaware of a full bladder, a lack of central inhibition is a common symptom of aging or following a stroke, resulting in urinary incontinence. Moreover, the pontine micturition center is in charge of our urination by completing the voiding task. Normally, the spinal micturition center (S2 and S3) at the conus medullaris comes from above (higher cortical centers). The spinal micturition center is active in infants until cortical inhibition develops, and it is usually inactive in adults except following a spinal injury when it can independently but ineffectively function [28]. Micturition is controlled in the upper center by spinal tracts in the intermediolateral white matter near the H-shaped gray matter. In sequence from medial to lateral, these tracts are (1) the autonomic motor, (2) the somatic motor, and (3) the all sensory tracts. Bladder distention induces low-level vesical afferent activity that stimulates the sympathetic outflow of the hypogastric nerve to the bladder outlet (the bladder base and the urethra) and the pudendal outflow to the external urethral sphincter. These are guarding reflexes that promote continence and are activated by spinal reflex pathways. Sympathetic nerve activation also inhibits detrusor muscle contraction and affects neurotransmission in the bladder ganglia. The rostral pons pontine storage center may stimulate striated urethral sphincter action. During urine evacuation, intense bladder-afferent activity in the pelvic nerve activates spinobulbospinal reflex pathways that go through the pontine micturition center, which causes an increase in parasympathetic outflow to the bladder and urethral smooth muscle while decreasing sympathetic and pudendal outflow to the urethral exit. Ascending afferent input from the spinal cord may travel through relay neurons in the periaqueductal gray (PAG) before reaching the pontine micturition center [29, 30] (Figure 1).

The neuromodulation method of action in OAB treatment is not understood. However, the therapy appears to modify spinal cord reflexes and brain involvement via afferent signals rather than direct motor stimulation of the detrusor or urethral sphincter. According to the most widely recognized explanation, neuromodulations interrupt or interfere with the afferent nerve input to the sacral spinal cord, limiting detrusor overactivity and resulting in clinical alleviation of urine frequency and urgency [31, 32]. Electrical stimulation of the pelvic nerve, pudendal nerve sensory fibers, or muscular nerve fibers from lower limbs like the posterior tibial nerve can inhibit spinal micturition centers [33, 34]. Fine needles are used to stimulate the Sanyinjiao point (SP-6), in which is located four fingers' breadth above the medial malleolus (SP-6). SP-6 is most likely to be the site of the tibial nerve that has been used to restore energetic harmony [35].

## 4. Neuroanatomy of Posterior Tibial Nerve

**Figure 2:**
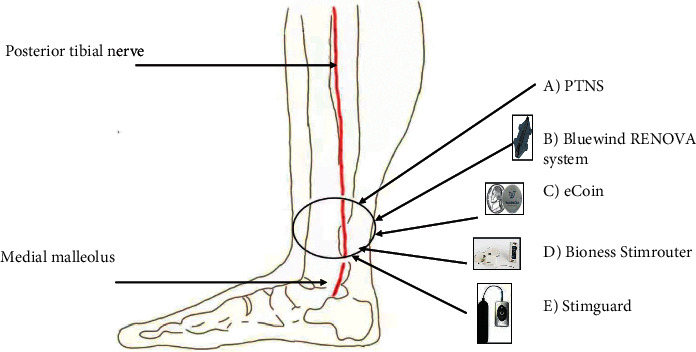
Posterior tibial nerve anatomy and site of different stimulation modalities. (A) The PTNS needle location; (B) BlueWind RENOVATM device; (C) eCoinTM device; (D) Bioness StimRouter; and (E) StimGuard.

Because of its tight relationship with the posterior tibial artery, the tibial nerve is also known as the posterior tibial nerve. It originated from the L4-S3 nerve roots composed of motor and sensory fibers; the pelvic floor muscle, bladder sphincters, and bladder detrusor muscle were supplied by the same root [36]. The tibial nerve is roughly three to four centimeters from the medial malleolus (halfway between the medial malleolus and the Achilles tendon) (Figure 2) [37].

## 5. Posterior Tibial Nerve Stimulation Classifications

### 5.1. Percutaneous PTNS

Following the FDA's recent approval of percutaneous PTNS for OAB treatment, which demonstrated its safety and efficacy, developments in percutaneous PTNS technology were visible. The Uroplasty, Inc. Minnetonka, MN urgent PC neuromodulation device acquired the conformity marking by the European area (CE) mark for OAB, and fecal incontinence is now commercially available. [17] A 34-gauge needle electrode is used that is implanted 4-5 cm superior to the medial malleolus to activate the tibial nerve [38], which is the same site that has been utilized in traditional Chinese acupuncture for the floor of pelvic disorder management and some pelvis organ dysfunction (SP6) [39].

The procedure of doing percutaneous PTNS can be performed at the outpatient clinic, and the patients were positioned supine or sitting, with their knees abducted and flexed and the soles of their feet together (“frog position”). A pad is put on the medial facet of the calcaneus on the same side as the grounding pad. The needle electrode is coupled to an external voltage source with a pulse generator (9 V). When the involuntary toe flexion or complete foot extension is linked with feeling in the ankle and sole, this is a good indication that the pulse is ideal. The toe flexion reflex is controlled by the S3 nerve root, which innervates the detrusor muscle. Depending on the patient's tolerance, a current level ranging from 0.5 to 9 mA at a fixed frequency of 20 Hz and a pulse width of 200 *μ*s is set and accordingly adjusted [40, 41]. Surprisingly, no defined treatment protocol exists. Different regimens (3, 6, 8, and 12 weeks) are discussed [35, 41–43]. The most often utilized percutaneous PTNS program involves weekly sessions lasting around 30 minutes for ten to twelve weeks [38].

Percutaneous PTNS is a relatively low-risk operation, with the most common consequences being mild bleeding, discomfort, and skin inflammation caused by the needle placement. Other uncommon side events include cramps of the leg, foot soreness, and a vasovagal response. Percutaneous PTNS, unlike SNM, avoids unpleasant impulses in the private area and the morbidity and cost associated with permanent implant placement [41].

### 5.2. Transcutaneous PTNS

The requirement for repeated stimulation sessions under physician supervision is a significant drawback to percutaneous PTNS, and recently, the COVID-19 pandemic is wreaking havoc on healthcare systems worldwide. Urology departments have fundamentally altered their daily practices in response to this new threat. Functional urology and pelvic floor disorders have been frequently deemed appropriate for a deferral during trying circumstances. The long-term consequences of this decline in functional urological clinical activity are unknown, but many patients may experience treatment delays, impairing their physical and psychological health and quality of life. Efforts should be undertaken to alleviate this patient group's burden without jeopardizing the safety of patients and healthcare professionals. [44] The transcutaneous PTNS approach can be safely performed by putting surface electrodes in the tibial nerve's innervations site; physiotherapists more typically utilize the latter because of its noninvasive nature. A recent study demonstrated that both the percutaneous PTNS and transcutaneous PTNS produce the same results when treating OAB and bowel dysfunction [45–48].

The self-adhesive ambulatory equipment, GEKO (Firstkind, Buckinghamshire, UK), was recently released. The tibial nerve is transcutaneously stimulated by a self-applying GEKO system that allows patients to freely move. It was designed at the beginning for deep venous thrombosis treatment [49]. A pilot trial was recently conducted to examine fecal incontinence results, and OAB [50, 51] participants in this open-label OAB trial were randomly assigned to receive 30-minute therapy sessions once daily or once weekly. The stimulation area was five cm superior to the medial malleolus, and the current was 27 mA, and a frequency of one Hz was set during treatment between 70 and 560 *μ*s, and the pulse width was increased (seven settings) [50] Seth et al. found a 53 percent response rate to the global response assessment (GRA) and International Consultations on Incontinence Questionnaires (ICIQ-OAB and ICIQ-LUTS). They found no significant variations among different groups in their three days voiding diary results (weekly treatment vs. daily). After three months of therapy, the combined group's leakage rate dropped to 1.3/24 h, compared to 2.5/24 h at baseline.

The noninvasive feature of the GEKO system and a significant proportion of client satisfaction in user-friendliness are its primary advantages [50]. The disadvantage of this technology is deceased efficacy due to skin impedance of stimulus and fixed-parameter settings. Moreover, improper placement of electrodes by patients may lead to ineffective results. Additional research on the efficacy of this approach in the OAB field is necessary to compare it with control and other treatment modalities [35, 52]. However, transcutaneous PTNS and percutaneous PTNS are effective and safe treatments for OAB in adult age; few randomized sham-controlled trials in a pediatric population have been conducted [53].

### 5.3. Posterior Tibial Nerve Implant Stimulation

While new technologies are being developed to treat OAB syndrome, we would like to focus on novel small implanted devices that stimulate the tibial nerve, providing physicians and patients with more treatment options when conservative measures fail to relieve symptoms [54]. This study will summarize different implant types and their efficacy and safety.

#### 5.3.1. The BlueWind RENOVA Apparatus

The BlueWind RENOVA device is a remote tibial nerve stimulation technology that does not require batteries that anyone can use. An implant, an external control unit (ECU), and a physician programmer are the three components that make up the system [55]. The pacemaker is a 25-millimeter graft with microscopic fixating wings that protect the device from moving and causing damage. The implantation is secured near the tibial nerve using an open surgery technique, while the patient is under local anesthetic. Following the recovery period, the clinician programmer then programs implant by ECU.

The clinician can choose a patient-specific treatment configuration according to the motor or sensory response to optimize the therapeutic outcome. It has a pulse width range of 50-800 milliseconds, a range of frequencies of up to 40 hertz, and an intensity range of 0–9 mA. Interestingly, the BlueWind RENOVA is a closed loop in which the patient can only wear the ECU during treatment sessions and freely move in between without restriction. The ECU supplied the implant with the energy required for treatment and altered the amplitude between the clinician-set minimum and maximum tolerated levels [56]. A first trial reported a response rate of 71% throughout a six-month Follow-Up (FU) period. One major adverse event associated with the technique happened, prompting the device's explanation. Three-year results show that, after 36 months following system activation, about 75% of patients are still responders in those who stayed in an extended study [57]. The closed-loop nature of the BlueWind RENOVA technology is an advantage. As far as we know, it is the only implantable device that makes use of a closed-loop structure to achieve that the energy provided from the ECU to the implant remains stable through treatment sessions. Additionally, the system has a lower migration risk due to the implant's suture holes and size. The open surgery technique to implant this system is the main drawback compared to percutaneous PTNS and other injectable implants [35].

#### 5.3.2. The Bioness StimRouter System

Initially, the Bioness StimRouter neuromodulation device has been designed for those who were suffering from chronic pain [58]. The instrument can be conveniently placed near the selected peripheral nerve using a local anesthetic [59]. The Bioness StimRouter is a custom-made lead transcutaneously powered by an external pulse generator (EPG). A receiver, electrodes, and an anchoring system are all included in the implanted lead [60]. During implantation, ultrasound or fluoroscopic imaging may aid with the stimulation probes' placement, which is used to test stimulation to ensure proper peripheral nerve targeting. Following confirmation of proper placement, the introducer set is positioned over the stimulation probe, and the probe is replaced with the stimulation lead. Finally, the lead's proximal end is subcutaneously buried 2 cm from EPG's intended location. [61]. Patients wear the EPG on their skin during tibial nerve stimulation and use a patient programmer for modifying parameter values and tracking usage [58]. The parameters employed in the chronic pain trial included phase durations ranging from 7 to 5 u/pulse rate ranging from 1 to 200 Hz and treatment times ranging from 10 minutes to 12 hours [59]. There were no device-related adverse events identified throughout the chronic pain research; however, five patients had their device removed due to dissatisfaction with its efficacy, one due to the development of chronic dermatitis/sensitivity to the electrode patch, and one due to lead rejection [59]. The StimRouter's advantage is the minimum invasive surgery required to implant the lead, which is constructed with anchors to prevent movement. There are only two minor incisions in the lower leg of patients. Another feature of this system is the ability to program the patient programmer with up to eight distinct treatment/stimulation regimens. As a result, treatment is tailored to the individual and is determined by the patient's choices. The disadvantage of the technology is the energy loss associated with the energy transfer via surface electrodes. Passively, the lead transfers energy from the superficial end (near to the EPG) to the electrodes. As a result, the ideal amplitude is likely to be 5-10 times greater in everyday practice than during test stimulation at implantation time [35]. There are currently no published studies on this neuromodulation technology in the OAB field. A multicenter, prospective, randomized, double-blind study is currently underway.

#### 5.3.3. eCoin

The eCoin is a revolutionary implantable neuromodulation technology (Valencia Technologies Corporation) that is based in Valencia, California. It is implanted using a less aggressive open surgery, in which a leadless and battery-powered device is placed into the inner aspect of the tibia under local anesthetic. The device is nickeled plated and housed in a titanium casing, which measures 23 mm in diameter and 2.4 mm in thickness [17, 62].

Following implantation, treatment sessions of 30 minutes each are automatically administered by the device every two days for a total of 12 weeks. Subsequently, the intensity of the therapy is lowered once every 15 days. However, the patient has no action to initiate a treatment session; the patient can use an external controller to change the amplitude from 0.5 to 15 mA. Other parameters are fixed: pulse width of 0.2 milliseconds and pulse rate of 20 milliseconds [63]. MacDiarmid et al. recently released the first six months' data (*N* = 46). A total of 67 percent of patients were classified as responders after six months' FU, as determined by their three-day voiding diary. Eleven patients were identified that had no further UUI events over the six months following FU. Patients reported a threefold improvement in I-QOL and PGI-I scores at the beginning and the half-year following FU treatment. Two instances of system explanation were documented by MacDiarmid et al. in relation to safety. There was only one patient who desired an explanation due to cellulitis and the second due to device migration 1 cm posterior, resulting in loss of stimulation [63].

A new prospective pivotal trial was conducted to assess the safety and efficacy of eCoin for the treatment of refractory UUI. A total of 133 of the 137 individuals enrolled received eCoin implants, whereas 132 were included in the intention-to-treat population. About 98% of the 132 individuals were women, the mean ± SD age was 63.9 ± 10.9 years, and the mean number of daily UUI episodes was 4.3 ± 3.1. The primary effectiveness analysis revealed that 68% (95 percent confidence interval (CI): (60%–76%) of patients experienced a 50% reduction in UUI episodes 48 weeks after activation; 16% of implanted subjects experienced device-related events through 52 weeks postimplantation. The authors conclude that eCoin proved clinical value when treating overactive bladder syndrome by automatically delivering an intermittent low-duty cycle and implanting it via a minimally invasive and quick procedure [64].

The advantage of this device is that the implantation technique is very simple and quick compared to SNM or other portable technologies. The battery-powered system might become advantageous because it will not require patient participation for function, resulting in 100 percent adherence. The disadvantage of the approach is that patients cannot increase the number of therapy sessions based on personal preferences or deteriorating symptoms. During maintenance therapy, the treatment session automatically begins every 15 days, which may also occur during a period when the patient is undesired to undertake treatment. Another drawback is the likelihood of migration, resulting in diminished tibial nerve stimulation. Furthermore, because it is battery powered, it must be replaced, which needs extra procedures for patients, similar to SNM, but more frequently due to its small size. On the other hand, long-term findings are required to establish efficacy and safety [35].

#### 5.3.4. StimGuard

Another implantable device is StimGuard LLC's minimally invasive chronic technology [24]. In this procedure, a patient receives an implanted stimulator with an embedded receiver through a 5 mm skin incision, and the energy supply for the implanted stimulator is a small, rechargeable transmitter worn by the patient. The external energy source is connected to external antennae and is worn close to the implant's internal antenna [35].

As a result, the system is an open loop, which means that the amount of energy given from the wearable to the electrode is stable, but it is not controlled secondary by an external device [24, 35]. In most cases, patients are told to use the implant overnight, with a maximum treatment time of eight hours permitted [65] Sievert et al. published the first results of this technique for two groups of patients, which was the first time this technique had been documented. Group 1 consisted of two male patients who had neurogenic lower urinary tract dysfunction and were included in the study (2014). The second group consisted of one male patient with spina bifida and five female patients with OAB (2016). Although both patients in the first group were completely dry two months following surgery, they were forced to quit because their medical conditions had worsened. In addition, migration of electrodes was observed in one patient. In the second group (*N* = 6), five patients showed significant improvement, with UUI episodes reducing from 2.1 per day at baseline to 2.5 per month three months after surgery. One male patient did not respond to treatment and was, therefore, excluded from the study as a result of his lack of improvement. The primary advantage of this technology is that it allows patients to receive treatment overnight, but electrode migration could be a significant issue [65]. Currently, a study is being undertaken to compare StimGuard CAN-stim against Medtronic InterStim [35, 54, 56, 57, 63].

## 6. Diagnosis of OAB

Depending on the clinical symptoms, including urgency and frequency (both day and night), with or without incontinence, it is possible to make a diagnosis of OAB. Patients with risk factors for urinary tract infection (UTI), bladder pathology, and stone disease should be screened for these disorders. Patients with a history of smoking may experience OAB symptoms due to bladder cancer, particularly carcinoma in situ, whereas bladder or distal ureteral stones may cause urgency and frequency. Indications of bladder pain syndrome (formerly known as interstitial cystitis) or pelvic floor dysfunction include frequent urination and nocturia that are accompanied by pain [66]. Each patient should have a full history taken, a physical examination performed, and a urinalysis performed. All symptoms, including the onset and duration of symptoms, the number of voids, the number of urgency episodes, and the number of incontinence occurrences per day, should be documented. The presence of concomitant symptoms such as dysuria, hematuria, visual abnormalities, muscle weakness, or paresthesia may point to a neurogenic diagnosis. When coughing or performing the Valsalva technique, there is a lot of leaking, indicating stress urinary incontinence. When discussing previous treatments (behavioral therapy, drugs, or pelvic surgery), it is crucial to remember any past tobacco, alcohol, or caffeine use. Prescription drugs may exacerbate OAB symptoms or combine with other treatment options; therefore, it is vital to ask about them [14, 67].

In addition to looking for neurological impairment, the physical examination should also search for musculoskeletal weakness or deformity. Women may experience vaginal atrophy, concomitant stress incontinence, and pelvic organ prolapse, which can be detected via a pelvic examination. Male examinations should be carried out to rule out the presence of an enlarged prostate, urethral illness, or infection in the male reproductive system. Lower extremity edema should be noted in these patients because they may need to empty their bladders more frequently at night due to fluid mobilization when supine. If you are experiencing excessive nocturia, you should consult your doctor about sleep apnea. Urine testing can detect infections or hematuria, both of which indicate the need for further investigation if they are detected [66].

When it comes to establishing the etiology of OAB, frequency-volume charts and bladder diaries are both effective tools. It is important that you keep account of the type and amount of fluid taken during each voiding event and any indicators of urgency or incontinence that you experience. In challenging patients such as those with neurological disease or those who have failed recurrent OAB treatment, procedures such as urodynamics, cystoscopy, kidney ultrasonography, and bladder ultrasonography should be reserved for them.

## 7. Management Outlines of PTNS

**Figure 3:**
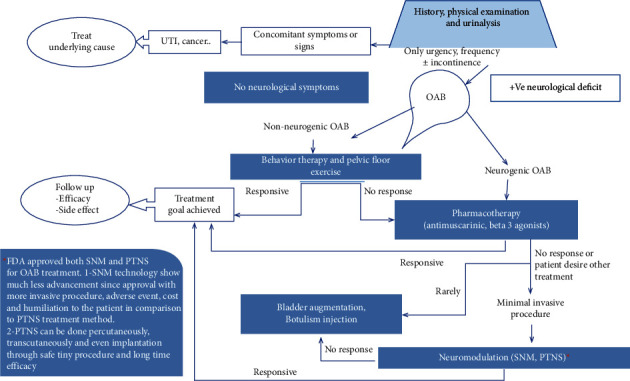
Diagram illustrating diagnosis of overactive bladder syndrome and role of posterior tibial nerve stimulation in its management. OAB: overactive bladder, UTI: urinary tract infection, SNM: sacral neuromodulation, PTNS: posterior tibial nerve stimulation, and FDA: Food and Drug Administration.

Patients complaining of urinary frequency (both daytime and night), and the urgency with or without incontinence must do urine analysis to detect UTI or any other obvious pathology. After exclusion of urinary tract-related problems, we can diagnose it as OAB. Moreover, we search for the accompanying symptoms and signs that may differentiate neurogenic from non-neurogenic OAB syndrome, in which treatment plans can be accordingly decided. PTNS emerges as one of the best modalities to treat this bothersome issue, either percutaneous, transcutaneous, or as an implant PTNS (Figure 3, Table 1).

The following section will discuss PTNS clinical applications and their role in a different category of functional lower urinary tract problems.

### 7.1. Neurogenic OAB

The incidence of neurogenic lower urinary tract dysfunction in people with MS is high, with a pooled prevalence of 68.41 percent using self-report measures and 63.95 percent using urodynamic examinations in people with MS. A small number of studies have been conducted to assess the potential efficacy of PTNS for the management of bladder storage symptoms in people with multiple sclerosis (MS). However, there has been little consensus on the optimal electrical stimulation parameters, including the frequency, duration, and the number of treatment sessions to be used [68].

Patients with Parkinson's disease are another category who suffer from OAB symptoms that necessitate long-term management that did not show adverse effects or interact with their medications. A PTNS can be effectively utilized in clinical practice to alleviate OAB symptoms in those with Parkinson's disease, and the improvement appears to remain at FU (30 and 90 days). [52, 69–73]. However, more future studies are still on their way to prove PTNS efficacy and safety in patients with Parkinson's disease.

Patients who complained of spinal cord disease either after acute trauma accidents or due to a pathologic process that ends with symptoms similar to OAB require, in addition to rehabilitation protocol to a safe, effective way of restoring and treating OAB symptoms. PTNS is a safe and feasible technique for acute traumatic spinal injury rehabilitation. Previous studies have shown that bladder capacity and an episode of detrusor sphincter dyssynergia dramatically deteriorated in the control group but remained stable in the PTNS group, implying that PTNS positively influences neurogenic course. [74–76].

### 7.2. Nonneurogenic OAB including Refractory OAB

Because OAB is a collection of urinary tract symptoms that is a chronic condition with a limited chance of cure, it is important to manage patient expectations. At the moment, the specific pathophysiology of OAB is unknown; however, it is believed that numerous causes contribute to this complex illness. Tibial nerve stimulation is an outpatient technique that temporarily modifies the tibial nerve that shares with detrusor muscle innervation [77].

PTNS can be safely used before invasive treatment in resistant OAB, and it was more effective if used three times a week than performing it once a week in refractory OAB patients [78–82].

### 7.3. Children

PTNS is an effective minimally invasive therapeutic option for children who do not respond to medical treatment for fluid volume deficit (FVD). PTNS significantly improves episode frequency, incontinence episodes, overall and daytime Dysfunctional Voiding Symptom Score (DVISS) ratings, and QoL scores. Treatment effectiveness is maintained even after the second year of treatment. [53, 83, 84] According to 12 sessions with patch electrodes, the PTNS approach was a good, safe, noninvasive, and long-term therapy option for this youngest category of patients.

## 8. Conclusions

Recently, advancements in technology have played an essential role in treating OAB by noninvasive continuous or semicontinuous posterior tibial nerve stimulation. Because the COVID-19 epidemic prevents these people from seeking medical attention, implantable PTNS is a practical and comfortable option for them. We talked about the history of tibial nerve stimulation and how it works on the micturition reflex at the spinal cord level. In addition, we discuss its neuromodulation role in the treatment of different kinds of OAB. Finally, we compared the various types of PTNS modalities in terms of advantages, technical concerns, safety, outcomes, and drawbacks. Compared to previous methods, the new implantable technologies show promising results in terms of efficacy, patient tolerance, and lowering the burden on physicians and the healthcare system. However, this result is based on short-term trials; therefore, additional research is required to demonstrate long-term and large-scale safety and efficacy.

## Figures and Tables

**Table 1:** Summary of some trials compared with different kinds of PTNS and other treatments for OAB.

First author (year)	Region	Study type	Study period	Age	Total number	Intervention	Comparison	Treatment cycle	Follow-up	Conclusion
Govier FE (2001)	USA	Clinical trial; prospective	2000-2001	57.4	53	Percutaneous PTNS	Baseline	Weekly	12 weeks	Reduction in mean daytime, nighttime voiding frequencies, and urge incontinence. Improvements in quality of life (QoL) indexes
Rostaminia G (2019)	USA	Retrospective	January 2011-December 2017	72.7	162	Percutaneous PTNS	Baseline	Once a week	12 weeks	Improvement in all three OAB symptoms and QoL
Jacomo RH (2020)	Brazil	Clinical trial	August 2017 to October 2018	68.62	50	Transcutaneous PTNS	Transcutaneous para sacral nerve stimulation (TPSNS)	Twice a week	4 weeks	Both groups' symptoms improved as measured by the ICIQ-OAB and ICIQSF, but TPTNS is more effective than TPSNS
Ramírez‐García I (2019)	Barcelona	Case control; retrospective	2015 and 2016	59.6	68	Transcutaneous PTNS	Percutaneous PTNS	Weekly	12 weeks	Both techniques lessened urgency incontinence, daytime frequency, and greatly improved the QoL
Padilha JF (2020)	Brazil.	Randomized controlled trial	2019-2020	>18 years	99	Transcutaneous PTNS	TPSNS and placebo	Weekly	12 weeks	NA
Martin-Garcia M (2019)	England	Randomized controlled trial	March 2015 and April 2016	Over 18 years	66	Transcutaneous PTNS	Percutaneous PTNS	Flexible home-based sessions	6 weeks, and 3 and 6 months	Similar positive result in both groups with less frequency, urgency, and improvement in QoL
Seth JH (2018)	England	Case control; retrospective	N\A	46.4	48	Transcutaneous PTNS	Baseline	Once daily or weekly	3, 8, 12, and 16 weeks	Significantly effective in the decrease of all OAB symptoms for treated patients
Ghijselings, L (2021)	Belgium	Randomized controlled trial	2021	5-12	N\A	Transcutaneous PTNS	Percutaneous PTNS and sham control	NA	12 weeks	TPTNS is significantly effective like PPTNS in the management of OAB symptoms
H.M.K (2017)	USA	Case control; retrospective	N\A	54	15	BlueWind Medical, Herzliya, Israel	Baseline	6 times per week	1 and 3 months	Safe, effective, and well-tolerated the modality in OAB patient treatment
Dorsthorst	—	Prospective	—	56.1	34	BlueWind RENOVA iStim system	Baseline	Automatic	36 months	The OAB symptoms significantly improved in treatment group.
MacDiarmid (2019)	USA and New Zealand	Prospective	—	63.4	46	eCoin	Baseline	Automated stimulation sessions	3 and 6 months	eCoin showed safe and effective method in the treatment of OAB
Alexandra Rogers (2021)	USA	Cohort study; prospective	August 14, 2018, to April 2, 2019	63.9	137	eCoin	Baseline	Automated stimulation sessions	48 weeks	eCoin demonstrated clinical benefits for treating overactive bladder

## Abbreviation

ICS: International Continence Society
UUI: Urgency urinary incontinence
QoL: Quality of life
SNM: Sacral nerve stimulation
PTNS: Posterior tibial nerve stimulation
NIH: National Institutes of Health
FDA: The USA Food and Drug Administration
NICE: National Institute for Health and Clinical Excellence
OAB: Overactive bladder
FU: Follow-up
COVID-19: Coronavirus disease 2019.
